# A step toward bio-inspired dental composites

**DOI:** 10.1080/26415275.2022.2150625

**Published:** 2023-01-19

**Authors:** Janine Tiu, Renan Belli, Ulrich Lohbauer

**Affiliations:** Friedrich-Alexander Universität Erlangen-Nürnberg (FAU), Zahnklinik 1 – Zahnerhaltung und Parodontologie, Forschungslabor für dentale Biomaterialien, Erlangen, Germany

**Keywords:** Composite materials, biomaterials, dental materials, mechanical properties, bioinspiration, R-curve

## Abstract

This feasibility study aimed to develop a new composite material of aligned glass flakes in a polymer resin matrix inspired by the biological composite nacre. The experimental composite was processed by an adapted method of pressing a glass flake and resin monomer system. By pressing and allowing the excess monomer to flow out, the long axis of the flakes was aligned. The resultant anisotropic composite with silanized and non-silanized glass flakes were subjected to fracture toughness tests. We observed increasing fracture toughness with increasing crack extension (Δ*a*) known as resistance curve (R-curve) behavior. Silanized specimens had higher stress intensity K_R_-Δ*a* over non-silanized specimens, whereas non-silanized specimens had a much lower Young’s modulus, and higher nonlinear plastic-elastic J_R_-Δ*a* R-curve. In comparison with conventional composites, flake-reinforced composites can sustain continued crack growth for more significant extensions. The primary toughening mechanism seen in flake-reinforced composites was crack deviation and crack branching. We produced an anisotropic model of glass flake-reinforced composite showing elevated toughening potential and a prominent R-curve effect. The feasibility of flake reinforcement of dental composites has been shown using a relatively efficient method. The use of a biomimetic, nacre-inspired reinforcement concept might guide further research toward improvement of dental restorative materials.

## Introduction

The current selection of dental restorative resin composites for computer-aided design and manufacturing (CAD/CAM) continues to expand with advances in technology. While restorative dentistry sets its bounds with mechanical, biological and esthetical demands, it is imperative that materials development does not stagnate. Instead, development should intently move toward replicating the mechanical properties of the natural tissues they aim to restore. As the human enamel is a highly textured structure composed of high aspect-ratio reinforcing units, the reinforcing concept is optimized for intraoral loading and maximum damage resistance. Nacre presents a similar concept that should guide us in development of more naturally inspired reinforcing concepts. At this stage, the three-dimensional replication of structures in the micrometric scale remains too high a feat for current CAD/CAM technologies. However, incremental strides can be taken to ensure progress toward bio-inspired materials. Innovative attempts are seen in polymer-infiltrated ceramic scaffolds [1,2]. While exhibiting Young’s modulus matching with dentin, these infiltrated structures fall short in resembling the structural organization and other critical mechanical behavior seen in natural dental tissues [3]. Hence, the basic mechanical principles are often overlooked. This includes the required arranged configuration of high aspect-ratio microstructural units responsible for complex crack-particulate interactions. Noteworthy innovation is seen with the inclusion of short glass fibers in dental resin composites [4]. The fibers offer an efficient means of inducing crack bridging toughening mechanisms similar to those seen in human enamel [5,6]. Prism orientation, especially in the inner region of the enamel layer (called Hunter-Schreger-Bands), account for effective energy absorption and toughening during crack propagation from the surface toward the dentin-enamel junction [7]. The orientation of high aspect-ratio elongated microstructural units, be it fibers in composites or crystal phases in glass-ceramics [8,9], provide the large-scale anisotropic architecture that leads growing cracks to deflect into high-energy-consuming shear loading (mode-II) states [10].

The forced redirection of advancing cracks toward unfavorable and tortuous paths are toughening strategies commonly seen in biomineralized materials with hierarchical high-ordered structures such as bone [11], shells [12] and dental tissues [7]. Ultimately, crack advance is met with increased resistance for continued extension. This is a highly aspired material property in synthetic structural biomedical materials graphically depicted as rising *Resistance curves* (or *R-curve*).

**Figure 1.**
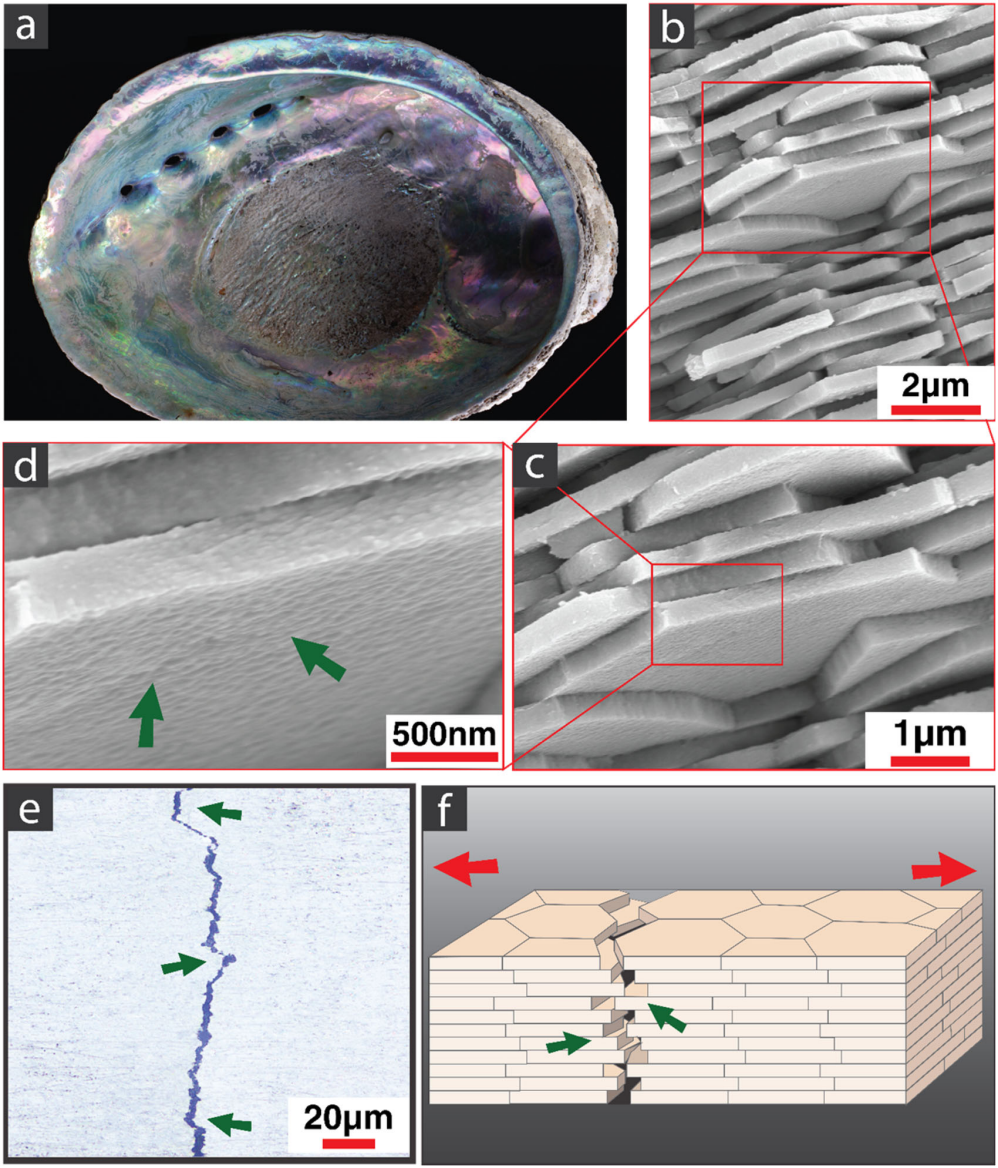
Structure of nacre; (a) natural nacre in an abalone shell (*Halitosis iris*); (b,c) aragonite platelets in brick-and-mortar structure; (d) arrows showing the nano-asperities on the surface of platelets providing a rough surface and increasing friction; (e) fracture pattern of nacre showing platelet pull out and bridging as well as crack deviations (arrows), more clearly seen in (f), schematic of nacre failure on the microscopic level.

Materials inspiration has been drawn from the inner layer of mollusk shells or nacre (Figure 1(a)). Nacre is composed of ordered interdigitated hexagonal aragonite platelets in a plastic interstitial organic matrix (Figure 1(b,c)). They form complex ‘brick-and-mortar’ structures that enable multiple toughening mechanisms to act simultaneously [13]. Notably, platelets in nacre interlock and slide on each other, bridging the crack wake and providing the energy dissipating processes at the microstructural level (Figure 1(e,f)) [14]. Surface nano-asperities on the platelets (Figure 1(d)) contribute by increasing frictional resistance [15], which adds to the bridging stresses that relieve the critical crack tip stress intensity factor needed for further crack advance. Meanwhile, the development of nacre-like artificial materials produced by a variety of techniques continues in engineering labs. These synthetic materials are capable of steep rising R-curves and reach saturation values for the applied stress intensity factor >8.0 MPa.m^0.5^ [16–18].

Here we demonstrate the feasibility of producing nacre-like architectures in experimental dental composites, leading to brick-and-mortar structures and substantial R-curve mechanical behavior. Combined with our current understanding of fiber-reinforced composites, we explore the nature of mechanistic processes in succession, from one-directional fibers to two-directional microstructural units. We introduce glass flake-reinforced dental composites for indirect CAD/CAM applications.

## Methods

Modified C-glass flakes (Glassflake Ltd. United Kingdom) with an average size of d_50_∼160µm and thickness 5 ± 1 µm (manufacturer information) silanized (methacryloxypropyltrimethoxysilane), and non-silanized were used as the ‘platelet’ fillers. The flakes were mixed and stirred into a monomer solution (60 wt.% urethane dimethacrylate (UDMA), 40 wt.% triethylene glycol dimethacrylate (TEGDMA) and heat initiator 1 wt.% benzoyl peroxide (BPO)) premixed at 3000 rpm/3 min (SpeedMixer™ DAC 150 SP, Hauschild, Germany). The mixture was placed in a vacuum chamber to remove residual porosity and slowly added to a custom CNC milled metal mold. The mixture was added in increments on a vibrating table to promote flake alignment until the mold was filled. The mixture was pressed at 80 MPa constant uniaxial pressure inside the mold while excess monomer flowed through the porous filter at the bottom. This method was based on a modified press assisted slip-casting method reported by Ekiz et al. [19], which induced flake alignment perpendicular to the pressing axis. The mold was tightened, placed in an oven (120 °C for 8 h) and slowly cooled overnight before the material release. Filler weight ratio was checked by combustion of the specimen at 500 °C for 1 h, where weight was checked before and after burnout.

Single-edge V-notch beam (SENB) specimens were prepared (2.5 mm × 5 mm × 25 mm) using a linear precision saw (IsoMet 5000, Buehler, USA), and the specimen surfaces were polished with decreasing grit sizes until 5 µm sandpaper or P4000 grit. A notch was created using a 1.5 mm diamond disk (length ∼2.3 mm) and sharpened with a sharp razor blade with 1 µm alumina paste to an additional 0.2 mm. Notches were measured under a light microscope (SteREO Discovery, V8, Zeiss, Germany). Specimens were gold sputter-coated before testing.

Fracture toughness tests were performed on a universal testing machine (Z2.5, Zwick/Roell, Germany) with an attached extensometer (LaserXtens, Zwick/Roell, Germany) using laser beam illumination following the digital speckle correlation method. Three-point bend (3-PB) configuration, according to ASTM E-1820 was performed with a crosshead speed of 0.01 mm/min. Specimens were loaded until significant deviance in compliance was observed from the load-displacement curve, at which point the test was manually interrupted. The specimen was observed under a light microscope (Leica TCS SL, Leica Microsystems GmbH, Germany) with an attached camera (AxioCam, Zeiss, Germany) to measure crack extension.

Successive applied stress intensity factors *K*_I_, were calculated for each crack extension:

(1) $$K_{I}=[\frac{PS}{BW^{\frac{3}{2}}}]f(a/W)$$

Where, *P,* is the maximum load, *S* is the support span, *B* is the thickness of the specimen, *W* is the width of the specimen, *a* is the total crack length, and $f(\frac{a}{W})$ for 3-PB is given by:

(2) $$f(\frac{a}{W})=\frac{3{\left(\frac{a}{W}\right)}^{1/2}[1.99-(\frac{a}{W})(1-\frac{a}{W})(2.15-3.93\frac{a}{W}+2.7{\left(\frac{a}{W}\right)}^{2})]}{2(1+2\frac{a}{W}){\left(1-\frac{a}{W}\right)}^{3/2}}$$

R-curves were also constructed from the energy perspective J-integral (*J*_R_). J-values at each crack length were measured in terms of plastic (*J*_pl_) and elastic (*J*_el_) contributions, their relationship is given by:

(3) $$J=J_{el}+J_{pl}$$

The energy partitioning was performed according to ASTM E1820 [20]:

(4) $$J_{I}=J_{\mathrm{el}}+J_{\mathrm{pl}}=\frac{K_{I}^{2}(1-v^{2})}{E}+\frac{\eta_{\mathrm{pl}}A_{\mathrm{pl}}}{B(W-a_{i})},$$

where *ν* is Poisson’s ratio (0.45) *E* is Young’s modulus (silanized = 26.9 GPa; non-silanized = 3.1 GPa) $A_{\mathrm{pl}}$ is the area under the plastic region of the load-displacement curve, *η*_pl_ is the plastic correction factor given in ASTM E1820 with the value 1.9, and $W-a_{i}$ is the uncracked ligament. The specimens were repeatedly reloaded and measured until crack extension was over 1.2 mm. Extensions over 0.7 mm are ASTM invalid for R-curve measurements. R-curves using linear-elastic (e.g. *K*_R_) or elastic-plastic principles (e.g*. J*_R_) were constructed against crack extension (Δ*a*). Microstructure and fracture surfaces were examined by gold sputter coating and imaging using scanning electron microscopy (SEM) (JSM-IT300LV, JEOL, USA; Auriga 0750, Zeiss, Germany).

## Results

**Figure 2.**
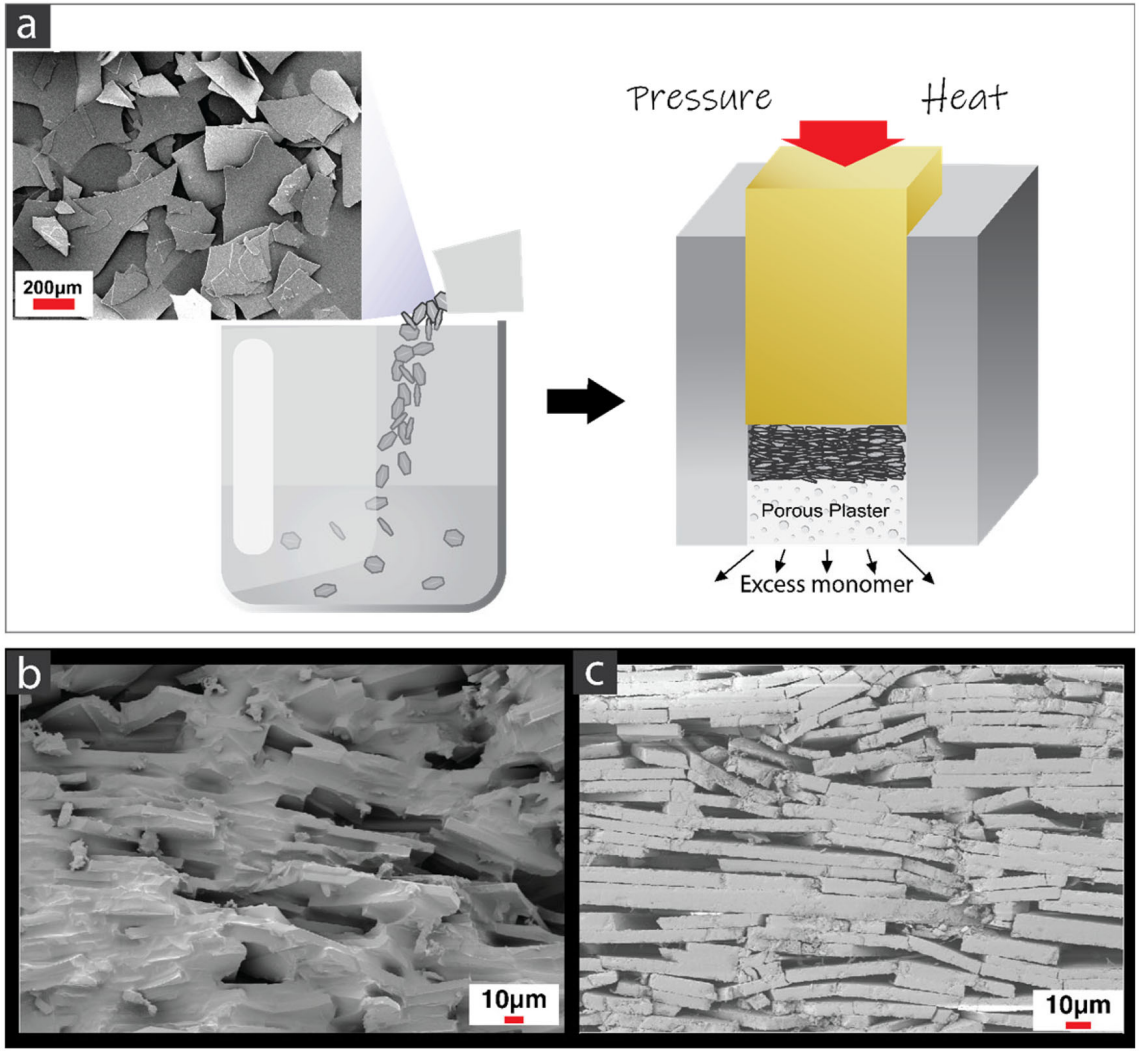
(a) Method of processing the experimental flake-reinforced composites; (b) SEM of fracture surface of the flake-reinforced composite; (c) SEM of surface after removing polymer.

Experimental flake-reinforced composites were produced using conventional dental monomers UDMA and TEGDMA, with glass flake fillers. SEM confirmed the alignment of the flakes. A representative fractured surface is shown in Figure 2(b), where both monomer and flakes are observed. Figure 2(c) shows the surface after removal of monomer after combustion (500 °C for 1 h).

**Figure 3.**
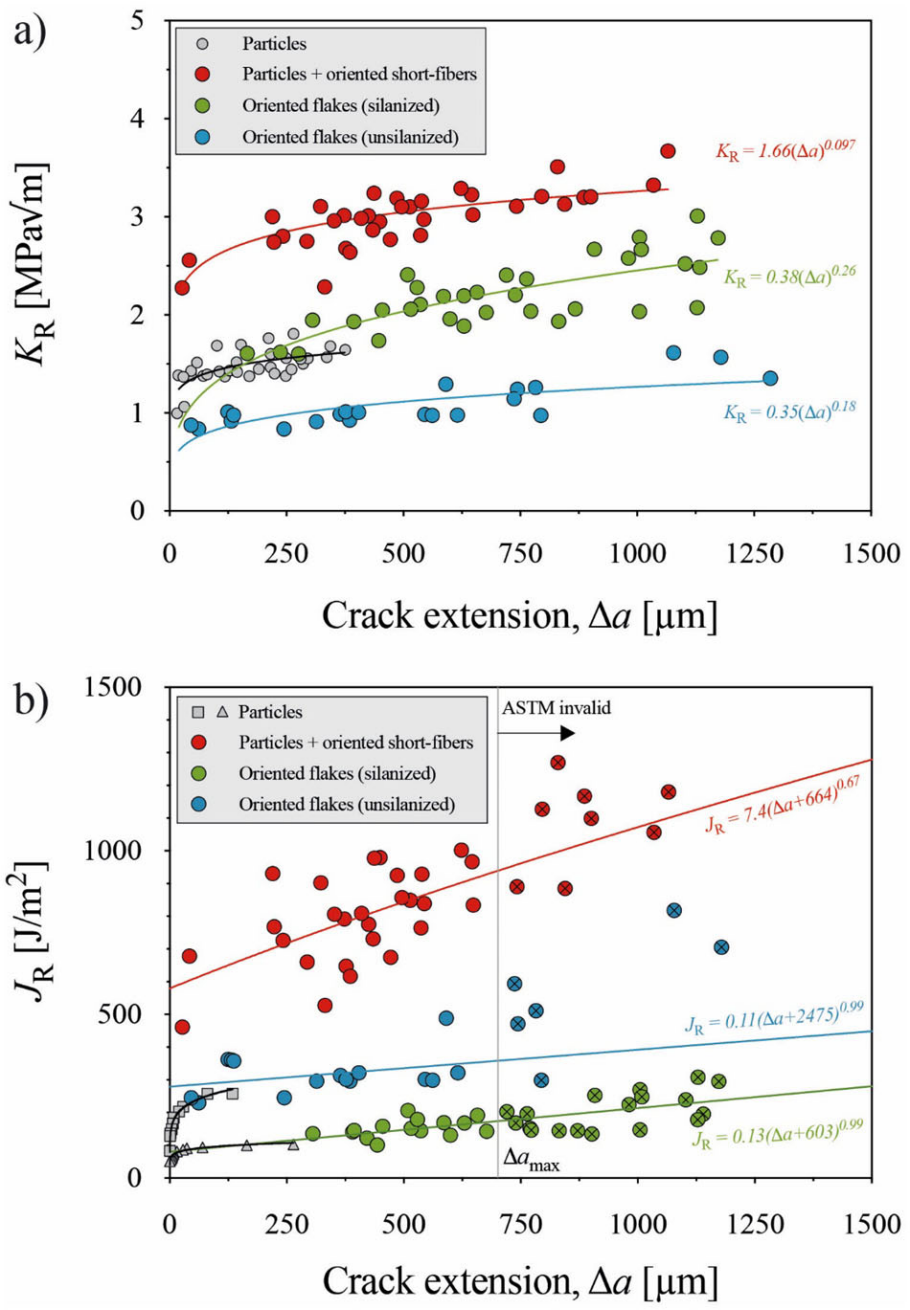
R-curves of silanized and non-silanized experimental flake-reinforced composites. Fracture toughness is shown in terms of stress intensity K_R_ (a) and nonlinear elastic fracture, J_R_ (b). Included were data on conventional composites (particles, grey dots):K_R_ data taken from Shah et al. [22] (see Figure 3(a)) and J_R_ data from Wendler et al. [6] and De Souza et al. [21] (see Figure 3(b)). Glass-reinforced composite data (particles + orientated short-fibers, red dots) K_R_ and J_R_ from Tiu et al. [5] were added.

**Figure 4.**
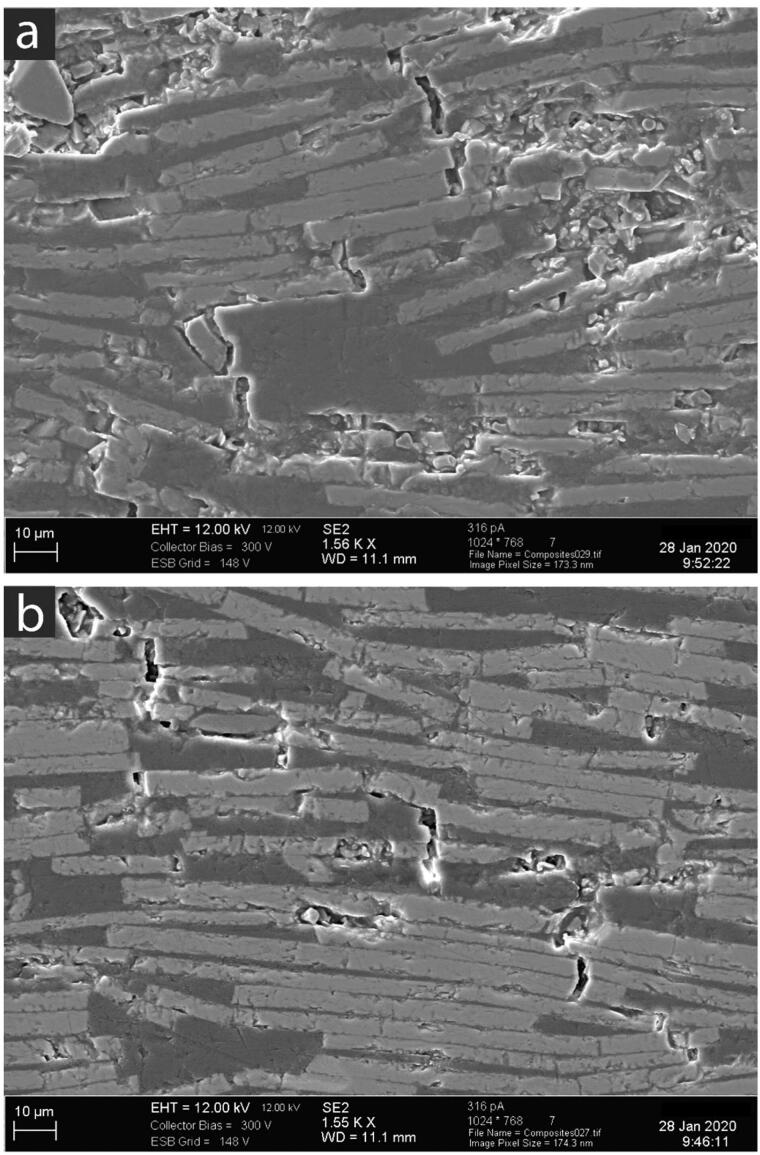
Micrographs of crack patterns along the surface of the flake-reinforced composites.

R-curves *K*_R_-Δ*a* and *J*_R_-Δ*a* for silanized and non-silanized specimens are shown in Figure 3. For comparison, we included measured R-curves from commercially available conventional composites [6,21,22] and commercially available aligned fiber-reinforced composites [5], both of which contain high particulate filler weight percentage. Both silanized and non-silanized specimens showed increasing R-curves, with non-silanized specimens showing a greater J_R_-Δ*a* over silanized specimens. SEM images of surface cracks are shown in Figure 4. Crack paths pointed out substantial deflection from the pure tensile direction and tended to move along the flake-matrix interface. At times the crack would extend around a flake and, in some areas, it traveled until it induced flake fracture or reached the extremity of a single flake.

## Discussion

The principal aim of this research was to develop a new composite material composed of glass flake fillers in a polymer resin matrix inspired by the brick-and-mortar structures of nacre. The method employed in this study allowed pressure input while allowing excess monomer flow. This forced flakes into alignment and increased filler percentage. The resulting composite block was anisotropic and matched dimensions for CAD/CAM processing, expanding the future clinical potential.

However, under similar processing pressure and heating protocol, vast differences were seen in silanized and non-silanized specimens. After burnout combustion, the filler weight percentage of silanized specimens was 81.26%, and non-silanized was 27.74%. Stark differences between the filler percentages are attributed to the hydrophobic non-silanized flake surfaces, whereas silanized surfaces increase wettability. The same behavior is also seen in fibers where silanized surfaces are seen to improve surface wetting and chemical adhesion [23]. Furthermore, it has been suggested the silane provides the mechanism of restrained layer theory, which suggests a balanced stress transfer between the high modulus fiber and resin matrix [24]. The restrained layer is certainly plausible in the case of flake-reinforced composites combined with flake percentage, responsible for the higher Young’s modulus values in silanized specimens (26.9 GPa)

One implication of a lower filler percentage, and consequently, a low Young’s modulus is reflected in the resulting R-curve. In *K*_R_-Δ*a* curves, silanized specimens have a more pronounced curve over non-silanized specimens. However, the inverse is true in *J*_R_-Δ*a* curves, where considerable energy is consumed during bending in non-silanized specimens, resulting in high *J*_el_. Remarkably, both silanized and non-silanized specimens can arrest crack growth over longer extensions, especially compared to conventional composites. The included values in Figure 3(a), taken from Shah et al. [22], show how conventional composites are able to develop toughening mechanisms starting from the same stress intensity range, but fail to continue the arrest over greater distances. In Figure 3(a), this is demonstrated for another conventional composite, which allowed controlled experiments up to 700 µm in crack extension, with unstable fracture thereafter. Although conventional composites demonstrate the onset of an R-curve effect, their ability to sustain controllable crack growth and crack arrest is limited to very small crack extensions, limiting the benefits of such behavior.

The increasing R-curves in flake-filed composites are more comparable to those of fiber-reinforced composites. Both fibers and flakes are high aspect-ratio microstructural units capable of inducing anisotropy when aligned. The aligned commercial composite with 25 wt.% of fibers and 45 wt.% particulates, has been demonstrated to increase resistance to crack growth significantly. Higher values in fiber-reinforced composites are shown in both K_R_-Δ*a* and J_R_-Δ*a* curves, even with a lower percentage of fibers than the flake percentage in silanized specimens. This is a consequence of a reinforced matrix system with particulates, as opposed to the unfilled matrix in our experimental specimens. In an aligned fiber-reinforced system, the crack front encounters a fiber aligned perpendicular to load direction. Generally, the mismatch in Young’s modulus and sufficiently weak interfacial bonding forces the crack to tilt out of plane and around the fiber circumference, or along the fiber length as it debonds. With continued crack growth, the debonded fiber will begin to pull out behind the crack wake, continuing its toughening contribution through the frictional surfaces between the fiber and matrix. The generated energy consumption of events over a process zone around the crack tip reduces the crack advancing forces felt at the crack tip. Over greater distances, the length of fibers bridging behind the crack increases until the crack opening is displaced enough for fiber bridges to degrade. The toughening mechanisms in flake-reinforced composites are markedly different. Crack advance in flake-reinforced composites is resisted by greater lateral distances from aligned glass flakes. Crack growth is more tortuous, and cracks deviate alongside the flake-resin interface in almost a ‘step-wise’ pattern (Figure 4). The crack is forced to deviate, meander, and in some cases, evidence of branching was seen. Unlike fibers, flakes do not pull out and bridge behind the wake; a toughening process zone is not seen. Instead, toughness is located at the crack tip through tortuous routes. Additionally, the effects of silanized flakes play an essential role in toughening. The significant contribution of crack deviation is easily redirected through the interface of the flake and matrix. Without silane, this path offers the path of least resistance. Apart from increasing the wettability and packing, the silanization of flakes offers a bonded interface for the growing crack to overcome, toughening the system. Still, as seen in Figure 4, the interface is weak enough to hinder flake cracking and straight – less energetic – crack trajectories.

Current dental restoratives lack intricate architectural designs that can significantly improve mechanical properties. This study showed that by adjusting to high aspect-ratio fillers and aligning the long axis perpendicular to the preferential crack growth plane, anisotropic fracture behavior could be fostered. The increasing R-curve behavior exceeds the benchmark for most dental materials, which do not show any significant toughness mechanisms to arrest advancing cracks. Apparently the toughening effect only accounts for a loading perpendicular to the reinforcement and hence the clinical loading szenario has to be taken into account when placing such a highly texturized material. The experimental composite under investigation however, is a significant step into bio-inspired designs in dentistry. Adopting toughening strategies and principles from natural composites opens new possibilities for developing more biologically oriented materials with competing performance. Future iterations through optimization may include particulate-reinforced resin matrices, different aspect-ratio flake fillers, or even a hybrid of fibers and flakes to take advantage of multiple toughening strategies. Material development - as shown here – aims to improve mechanical resistance against clinical fracture. On the other side, material selection by the dentist and acceptance by the patient is also highly influenced by the esthetic appearance and might decide for the economic success of a final product. Whatever innovative dental material befalls us should continue to take a similar path of biomimetic strategies.

## Conclusions

This study demonstrates the high aspect-ratio glass flake fillers aligned in a resin matrix, producing a composite material showing anisotropy and increasing R-curve. The methodology employed in the study allowed for the processing of a composite which mimics the structure brick-and-mortar structure of nacre. The flake-reinforced composite deviates advancing cracks providing significant energy-consuming processes for increasing R-curve behavior, though in a different mechanism to that of fibers. This work highlights the case for aligned flake-reinforced composites in dentistry and is a potential way forward in dental restorative material development toward structural biomimetic restorations.
